# Updates in Kidney Transplantation From the 2022 Banff-Canadian Society of Transplantation Joint Meeting: Conference Report

**DOI:** 10.1177/20543581231209185

**Published:** 2023-11-13

**Authors:** Ian Carrigan, Sunita Mathur, Nicholas Bourgeois, Mélanie Dieudé, Daniel Fantus, Patricia Gongal, Anne Halpin, Alim Hirji, Holly Mansell, Caroline Piotrowski, Ruth Sapir-Pichhadze, Amanda J. Vinson

**Affiliations:** 1 Division of Nephrology, Department of Medicine, Dalhousie University, Halifax, NS, Canada; 2 School of Rehabilitation Therapy, Queen’s University, Kingston, ON, Canada; 3 Centre Hospitalier de l’Université de Montréal, Montréal, QC, Canada; 4 Department of Microbiology, Infectious Diseases, and Immunology, Faculty of Medicine, Université de Montréal, Montréal, QC, Canada; 5 Centre de Recherche de Centre Hospitalier de l’Université de Montréal, Montréal, QC, Canada; 6 Division of Nephrology, Department of Medicine, Centre Hospitalier de l’Université de Montréal, Montréal, QC, Canada; 7 Canadian Donation and Transplantation Research Program, Alberta Transplant Institute, University of Alberta, Edmonton, AB, Canada; 8 Alberta Precision Laboratories, Department of Laboratory Medicine and Pathology, University of Alberta, Edmonton, AB, Canada; 9 Division of Pulmonary Medicine, Department of Medicine, University of Alberta, Edmonton, AB, Canada; 10 College of Pharmacy and Nutrition, University of Saskatchewan, Saskatoon, SK, Canada; 11 Department of Community Health Sciences, Max Rady College of Medicine, University of Manitoba, Winnipeg, MB, Canada; 12 Department of Medicine, Division of Experimental Medicine, McGill University, Montréal, QC, Canada

**Keywords:** kidney transplantation, COVID-19, medical legislation, solid organ transplantation, Canada

## Abstract

**Purpose of the Conference:**

The 2022 Banff-Canadian Society of Transplantation Meeting in Banff, Alberta, brought together transplant professionals to review new developments across various aspects of solid organ transplantation (SOT) in Canada.

**Sources of Information:**

Presentations included consensus recommendations from expert-led forums; experiences with new procedures and legislation; reports from public health data repositories; original clinical and laboratory research; and industry updates regarding novel technologies. Speakers referenced articles and reports published in peer-reviewed journals and online, and unpublished data and preliminary findings.

**Methods:**

All authors attended presentations in-person or virtually. Recordings of select presentations were available for later review. Summaries emphasize concepts indicated by speakers as new and clinically relevant.

**Key Findings:**

The COVID-19 pandemic disproportionately affected solid organ transplant recipients (SOTRs), who experience worse outcomes of COVID-19 infection than the general population. Vaccinations demonstrate an attenuated immunological response in SOTRs yet provide meaningful protection. Monoclonal antibodies are effective for both passive immunization and treatment of COVID-19 in SOTRs. Infection control protocols have driven the development of virtual methods for clinical research, such as using home blood draws and virtual follow-up to evaluate vaccine efficacy in SOTRs; and patient care delivery, such as employing telerehabilitation post transplant. Access to living kidney donation is limited by various disincentives experienced by potential donors, which may be overcome by more efficient evaluations including a One-Day Living Kidney Donor Assessment Clinic. The International Donation and Transplantation Legislative and Policy Forum provided a means of establishing consensus guidance for organ donation and transplantation (ODT) program policy to standardize delivery across jurisdictions. The implementation of a deemed consent model for organ and tissue donation in Nova Scotia may provide insight as to whether this model indeed improves access to organs. Canada’s Indigenous population experiences unique barriers to transplantation, prompting efforts for more inclusive ODT policy-making. The Pan-Canadian ODT Data and Performance Reporting System Project has defined performance quality indicators, of which iTransplant and other point-of-care software solutions may facilitate collection; however, these endeavors ultimately require information technology infrastructure that exceeds the capabilities of the existing Canadian Organ Replacement Register and Canadian Transplant Registry. Pig-to-human xenotransplantation requires genetic modification of pigs and xenoantibody testing in recipients but may yet prove viable. Serum cell-free DNA, urine biomarkers, and genetic markers offer an alternative to routine biopsy for identifying subclinical rejection. Modified perfusion temperatures and perfusion solutions with hydrogen sulfide donor compounds may improve organ preservation. Molecular compatibility tools provide another means of improving SOTR outcomes, and the Genome Canada Transplant Consortium has been examining important considerations of their implementation.

**Limitations:**

We were unable to capture all presentations and topics at the meeting due to the sizable quantity and variety. Topics ultimately excluded from this summary include those in pathology including Banff Classification updates; those unique to extra-renal SOT; as well as numerous abstract and poster presentations, allied health provider forums, and business meetings. A portion of the material was presented by speakers prior to peer-review or publication.

**Implications:**

The various conference presentations summarized in this report identify methods by which individual clinicians and provincial ODT programs may improve access, delivery, and quality of SOT care in Canada, while additionally identifying gaps in the literature that investigators are encouraged to pursue.

## Abrege

**Objectifs de la conférence:**

En 2022, le congrès annuel de la Société canadienne de transplantation qui s’est tenu à Banff (Alberta) a réuni des professionnels de la transplantation qui se sont penchés sur les nouveaux développements dans divers aspects de la transplantation d’organes solides (TOS) au Canada.

**Sources:**

Les présentations portaient notamment sur : les recommandations consensuelles issues de forums dirigés par des experts; l’expérience avec les nouvelles procédures et lois; des rapports provenant de dépôts de données de santé publique; des recherches cliniques et des recherches de laboratoire originales; et les mises à jour du secteur sur les nouvelles technologies. Les intervenants ont fait référence à des articles et rapports publiés en ligne et dans des revues évaluées par les pairs, ainsi qu’à des données non publiées et des conclusions préliminaires.

**Méthodologie:**

Tous les auteurs ont assisté aux présentations en personne ou virtuellement. Les enregistrements de certaines présentations étaient disponibles pour visionnement ultérieur. Les résumés mettent l’accent sur les concepts jugés comme nouveaux et cliniquement pertinents par les intervenants.

**Principaux résultats:**

La pandémie de COVID-19 a affecté les receveurs d’une transplantation d’organe solide (RTOS) de manière disproportionnée; ces derniers ayant suivi une évolution plus défavorable que la population générale à la suite d’une infection à la COVID-19. La vaccination, bien qu’elle offre une protection significative, montre une réponse immunologique plus faible chez les RTOS. Les anticorps monoclonaux sont efficaces à la fois pour l’immunization passive et le traitement de la COVID-19 chez les RTOS. Les protocoles de contrôle des infections ont mené au développement de méthodes virtuelles pour la recherche clinique (p. ex. prélèvements sanguins à domicile, suivi virtuel pour évaluer l’efficacité du vaccin chez les RTOS) et la prestation de soins aux patients (p. ex. rééducation à distance) après la transplantation. L’accès au don de rein vivant est limité par divers facteurs dissuasifs pour les donneurs potentiels, mais ces obstacles peuvent être surmontés par des évaluations plus efficaces, notamment par une clinique d’un jour pour évaluer la candidature des donneurs vivants de rein. Le Forum législatif et politique international sur le don et la transplantation a fourni un moyen d’établir des lignes directrices consensuelles pour la politique du program de dons d’organes et de transplantation (DOT), dans l’objectif de normaliser la prestation d’une juridiction à l’autre. La mise en œuvre en Nouvelle-Écosse du consentement présumé pour le don d’organes et de tissus pourrait aider à déterminer si un tel modèle améliore effectivement l’accès aux organes. Les populations autochtones du Canada sont confrontées à des obstacles uniques en matière de transplantation, ce qui encourage les efforts visant l’élaboration de politiques plus inclusives en matière de DOT. Le Projet de système pancanadien de données et de mesure de la performance pour les DOT a défini des indicateurs de performance, dont iTransplant et d’autres solutions logicielles pour les points de soins, qui peuvent faciliter la collecte des données; ces derniers nécessitent toutefois une infrastructure informatique qui dépasse les capacités du Registre canadien des insuffisances et des transplantations d’organes et du Registre canadien de transplantation. La xénogreffe de porc à humain requiert une modification génétique des porcs et le dépistage de xénoanticorps chez les receveurs, mais elle peut encore s’avérer viable. L’ADN acellulaire sérique, les biomarqueurs urinaires et les marqueurs génétiques offrent une alternative à la biopsie de routine pour identifier les rejets subcliniques. Des températures de perfusion modifiées et des solutions de perfusion contenant des composés générateurs de sulfure d’hydrogène peuvent améliorer la conservation des organes. Les outils de compatibilité moléculaire offrent un autre moyen d’améliorer les résultats des RTOS, et le Genome Canada Transplant Consortium a examiné les aspects importants à prendre en considération pour leur mise en œuvre.

**Limites:**

Nous n’avons pas été en mesure d’assister à toutes les présentations en raison du grand nombre et de la grande diversité des sujets abordés. Les sujets exclus de ce résumé incluent la pathologie, notamment les mises à jour de la classification Banff; les sujets propres à la TOS extrarénale; ainsi que de nombreux résumés et présentations d’affiches, forums de prestataires de soins de santé alliés et réunions d’affaires. Une partie du matériel présenté l’a été avant l’examen par les pairs ou la publication.

**Conclusion:**

Les présentations du congrès qui sont résumées dans le présent rapport identifient les méthodes que les programs provinciaux de DOT et les cliniciens pourraient employer pour améliorer l’accès, la prestation et la qualité des soins en TOS au Canada, et soulignent des lacunes dans la littérature que les chercheurs sont encouragés à creuser.

## Purpose of the Conference

In autumn of 2022, the Canadian Society of Transplantation (CST) partnered with the Banff Foundation for Allograft Pathology to host the 2022 Banff-CST Joint Meeting in Banff, Alberta, Canada.^1^ This conference brought together physicians, pharmacists, nurses, physiotherapists, transplant coordinators, laboratory scientists, social scientists, and other transplant professionals for a series of virtual and in-person events. In this report, we aim to summarize and disseminate key concepts in clinical solid organ transplantation (SOT) and kidney transplantation specifically as presented within the core program of the 2022 Banff-CST Joint Meeting.

## Sources of Information

The conference was comprised of a series of plenaries each with a particular theme. Presentations within plenaries included consensus recommendations from expert-led forums and panels; first-hand clinician experiences with conventional practices and new procedures and legislation; reports generated from public health data repositories; original clinical and laboratory research; and industry updates regarding the development and real-world implementation of novel technologies. Speakers referenced articles and reports published in peer-reviewed journals and online databases; as well as unpublished data and preliminary findings from projects for which they are contributors.

## Methods

All authors were participants at the conference and attended in-person and/or virtually, with recordings of presentations available via a web-based application (Whova; San Diego, California) for later review. Summaries were composed directly from select presentations, supplemented by published works referenced within the presentations where available, emphasizing concepts indicated by speakers as new and significant. Key findings are presented in subsections that mirror to the plenaries in which the topics were presented with some modifications, including combining topics in Indigenous health into a single subsection (i.e., 5. First Nations, Inuit, and Métis Considerations) that was not itself a conference plenary.

## Key Findings

### COVID-19 and Transplantation

Immunosuppressed solid organ transplant recipients (SOTRs) have been disproportionately afflicted by the COVID-19 pandemic. COVID-19-attributable mortality rates amongst SOTRs early in the pandemic varied widely between jurisdictions but often exceeded 20%.^2,3^ The identification of steroid therapy, interleukin (IL)-6 inhibitors, novel antivirals, and monoclonal antibodies by 2021 reduced crude mortality rates among SOTRs, but they remain unacceptably high.^4^ Vaccination revolutionized the severe acute respiratory syndrome coronavirus 2 (SARS-CoV-2) risk in the general population, but SOTRs were not afforded the same benefit. Solid organ transplant recipients achieved an attenuated immunologic response to COVID-19 vaccination with neutralizing antibodies against wild-type SARS-CoV-2 present in 56.9%, against delta in 45.1%, and against omicron BA.1 in only 15.7% of SOTRs 3 months after a third vaccine dose.^5^ However, when breakthrough COVID-19 infections occurred, vaccinated SOTRs experienced lower hospitalization rates and, if hospitalized, shorter lengths of stay as compared to unvaccinated SOTR controls.^6^ These findings highlight the importance of establishing clinical, not just immunologic, COVID-19 vaccine efficacy among SOTRs.

Passive immunization may provide a means of protecting at-risk SOTRs. The PROVENT randomized controlled trial examined cilgavimab/tixagevimab (Evusheld), a preparation of long-acting monoclonal antibodies against the spike protein of SARS-CoV-2 that blocks viral entry into cells, as pre-exposure prophylaxis.^7^ Cilgavimab/tixagevimab decreased the incidence of symptomatic COVID-19 in the unvaccinated general population (pre-omicron variant) from 1% to 0.2% with minimal adverse effects. The efficacy of cilgavimab/tixagevimab in the transplant population and for the omicron variants is less established. Some studies have demonstrated relatively high breakthrough infection, hospitalization, and intensive care unit admission rates among kidney transplant recipients receiving cilgavimab/tixagevimab (9.4%, 35.9%, and 7.7%, respectively).^8^ Variability in efficacy, particularly during the omicron wave, has contributed to inconsistencies in recommendations regarding the use of cilgavimab/tixagevimab in SOTRs across Canada.^9^

The treatment of SOTRs with COVID-19 is evolving. Monoclonal antibodies have provided benefit for SOTRs with COVID-19, yet activity against omicron (bamlanivimab-etesevimab, casirivimab-imdevimab) and the BA.2 subvariant (sotrovimab) has waned significantly, and these agents are no longer routinely used.^4^ The management of symptomatic COVID-19 infection in SOTRs included monoclonal antibodies with efficacy against omicron (e.g. bebtelovimab in the United States) as a first-line agent in combination with dexamethasone and early remdesivir. While emergency use authorization for bebtelovimab has since been revoked by the FDA because of lack of susceptibility of recent variants,^10^ the latter 2 therapies remain the mainstay treatment for hospitalized immunocompetent and immunocompromised patients alike with severe COVID-19 infection.

Among potential donors, SARS-CoV-2 positivity poses an ongoing challenge due to uncertainty, with widespread discard of organs from deceased donors with COVID-19.^11^ An early study reported donor-derived COVID-19 infection in 3 lung transplant recipients, 1 of whom died from COVID-19-related complications.^12^ The donors were nucleic antigen test (NAT) negative for SARS-CoV-2 from the upper respiratory tract, but NAT positive from the lower respiratory tract. Interestingly, no nonlung organ recipients developed COVID-19 infection, prompting the cautious utilization of donors with lower respiratory tract SARS-CoV-2 NAT positivity for liver, kidney, and heart transplantation. A large, multicenter study of SARS-CoV-2 NAT positive donors demonstrated better quality organs and no statistical difference in graft failure or patient survival at 30 days for any of kidney, liver, or heart recipients as compared to NAT negative controls,^13^ and a smaller study demonstrated similar outcomes at 6 months of follow-up.^14^ The risk of transmission from SARS-CoV-2 positive donors appears to be low, but is unknown, with no long-term data available.

### New Realities in Patient-Care Delivery

Infection control protocols brought on by the COVID-19 pandemic have driven the rapid development of virtual methods for research and patient care delivery.^15^ Investigators aiming to assess the antibody response of COVID-19 vaccination among SOTRs successfully recruited participants remotely using social media.^16,17^ Participants underwent blood draws at home using TAPII devices (YourBio Health; Medford, Massachusetts); samples were delivered to laboratories using postal services; and virtual follow-up was completed to track clinical outcomes. Data for this cohort continue to be collected and have contributed substantially to the evidence base for COVID-19 vaccination among SOTRs, particularly highlighting limited immunogenicity in this population. Telerehabilitation after SOT has been widely adapted in place of in-person rehabilitation.^18,19^ Numerous barriers to delivery of telerehabilitation exist including limited patient access to technology and exercise equipment, and the need for remote monitoring. While safety and efficacy relative to conventional rehabilitation has not been established, initial studies have demonstrated generally favorable outcomes. Though born out of necessity, virtual approaches have proven effective and efficient and are likely to persist even with the ongoing easing of COVID-19-related restrictions.

Aside from pandemic-related pressures, health care delivery has needed to adapt to an aging Canadian population. Among SOTRs, frailty (i.e., a combination of physical frailty, sarcopenia, immune dysfunction, and multimorbidity) has been associated with delayed graft function, persistent disability, and mortality across organ types.^20,21^ However, frailty improves in SOTRs after transplant, and those who are most frail pretransplant experience higher degrees of improvement in health-related quality of life post transplant.^22^ Routine assessment for frailty indicators using a novel solid organ frailty index may allow for the identification of the most at-risk patients,^23^ and a comprehensive intervention consisting of rehabilitation and shared decision-making may lead to improved long-term outcomes for SOTRs experiencing frailty.^24^

### Barriers to Living Kidney Donation

Multilevel inefficiencies within the organization of kidney care impede access to living kidney donation.^25^ Kidney care in Canada is delivered by independent regional organizations that lack unifying mandates, funding models, and leadership. Within Ontario, the Ontario Renal Network (ORN) oversees the delivery of chronic kidney disease (CKD) and dialysis care while the Trillium Gift of Life Network (TGLN) oversees deceased kidney donation and transplantation. Living kidney donation is not formally managed by either organization, leading to potentially uncoordinated and ineffective efforts to recruit nondirected anonymous donors (NDADs), standardize pretransplant and posttransplant care, and provide patient education, including strategies to identify potential living donors.

In completing evaluation for eligibility and subsequently following through with donation, living kidney donors must overcome many financial and nonfinancial disincentives (Table 1).^25,26^ The median out-of-pocket expense incurred by Canadian living kidney donors was found to be $2217 (Canadian dollars) and exceeded $5500 for at least 25% of donors, despite most Canadian reimbursement programs offering a maximum payout of $5500.^27^ Reimbursement programs may be improved by expanding promotional efforts; simplifying paperwork required for the enrolment process; and increasing payout to better reflect financial disincentives, nonfinancial disincentives, and long-term dialysis savings related to improved recipient outcomes.

**Table 1.** Disincentives to living kidney donation in Canada.^26^

Financial disincentives	Nonfinancial disincentives
Travel to a transplant center	Excessive time commitment
Lodging near a transplant center	Decreased long-term quality of life
Loss of income while completing workup and recovering from surgery	Risk of death and other medical complications of nephrectomy
Home and dependent care	Inability to donate a kidney to a friend or family member in the future

**Figure 1.**
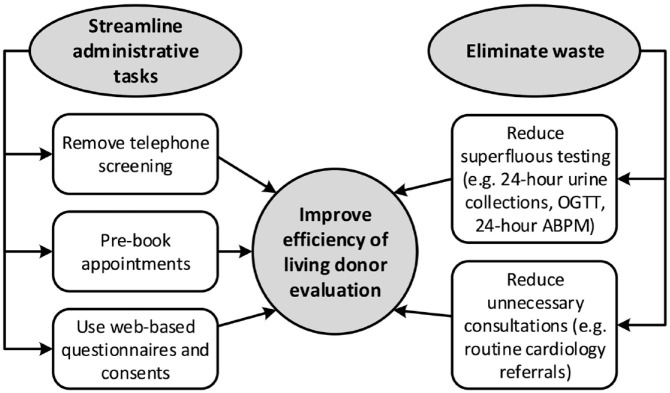
Methods to improve efficiency in living kidney donor evaluations.^27^ *Note.* OGTT = oral glucose tolerance testing.

There is no national benchmark for what is considered a reasonable timeframe within which to complete an evaluation for living kidney donation.^25^ Protracted evaluations lead to donors dropping out in response to fatigue and anxiety, among other disincentives. In Ontario, the median time between initial living donor contact and donation was 10.5 months.^26^ Multiple methods to optimize living kidney donor evaluations have been suggested (Figure 1). In Hamilton, Ontario, a One-Day Living Kidney Donor Assessment Clinic was piloted whereby most components of the donor evaluation (e.g., laboratory investigations; imaging; cardiac testing; and social work, nephrology, and surgery consultations) are completed within a single day.^28^ Since inception, more than 150 donors have been evaluated at this clinic, corresponding to approximately 4 per month and 25% of all living kidney donor evaluations at their center. Evaluation of patient outcomes is ongoing, but preliminary feedback regarding patient experience has been overwhelmingly positive.

### ODT Legislation and Presumed Consent

**Figure 2.**
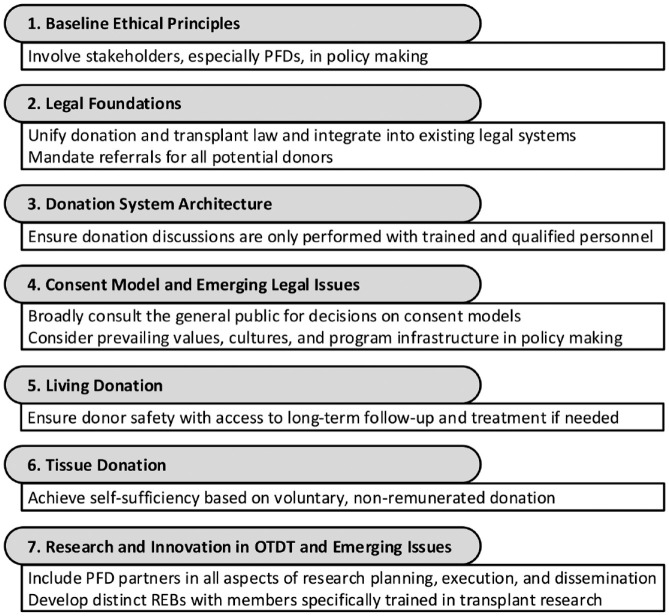
Domains for ODT policy reform as identified by the 2021 International Donation and Transplantation Legislative and Policy Forum, with select examples of proposed recommendations.^31,32^ *Note.* ODT = organ donation and transplantation; PFDs = patients, families, and donors; REBs = research ethics boards.

Organ donation and transplantation (ODT) systems depend on coordination between care providers and legislation, with marked variability in practices across jurisdictions. In October 2021, the International Donation and Transplantation Legislative and Policy Forum was held in Montréal, Québec, with a goal of developing expert consensus guidance that links evidence and ethical concepts to ODT legislative and policy reform.^29,30^ In the months preceding the forum, world experts in SOT were recruited and divided into groups each tasked with drafting consensus recommendations on topics within 1 of 7 domains (Figure 2). These recommendations are presently being finalized through engaging stakeholders in preparation for publication and knowledge translation.

Nova Scotia, Canada became the first jurisdiction in North America to introduce a deemed consent model for organ and tissue donation. The Human Organ and Tissue Donation Act (HOTDA) was passed in April 2019 and enacted January 18, 2021.^31^ Under this legislation, all persons who have not registered a donation decision are viewed as having consented to donate all organs and tissues after death. Next of kin may decline donation if they believe it is not consistent with the potential donor’s last known wishes. Several groups were excluded from this legislation including persons under 19 years of age; persons who lack capacity to make a decision regarding donation; and persons who are not permanent residents of Nova Scotia. Nova Scotia had been the highest performing Canadian province with respect to deceased organ donation in the early 2000s, but with rates having stagnated in the 2010s, legislators hope this model will offer successes observed in Spain and other jurisdictions with high-performing deceased donor programs where deemed consent is in effect.^32^ After HOTDA was passed, Nova Scotia’s provincial ODT program Legacy of Life faced a major infrastructure overhaul to support its enactment including onboarding a greater number of donor physicians and transplant coordinators, creating new roles including a dedicated quality database analyst and health care provider education lead, and developing new software including a new potential donor audit tool. Outcomes from 2020 and 2021 after passing HODTA, such as the number of donors per million population, have been variable, heavily confounded by COVID-19-related issues.^33^ It is expected that markers of success (e.g., donation rates and clinical outcomes for waitlisted patients) will be years in the making, and it will be challenging to distinguish whether such markers are attributable to the legislation itself or the associated infrastructure reforms within Legacy of Life.

### First Nations, Inuit, and Métis Considerations

The Indigenous population of Canada comprising First Nations, Inuit, and Métis (FNIM) peoples experience poorer health outcomes than the general population with higher rates of acute and long-term illness.^25,34,35^ Contributing factors include intergenerational impacts of colonialism leading to poverty, lower education, and food and housing insecurities, further confounded by jurisdictional complexities regarding the funding and provision of health care services for FNIM patients. Métis and nonstatus and off-reserve First Nations individuals receive services directly from the provinces or territories; however, status- and on-reserve First Nations individuals and Inuit receive supplementary federal funding through Indigenous Services Canada, which also directly provides health care services to First Nations and Inuit communities.^36^ This fragmented infrastructure results in gaps in care for FNIM patients who fall outside the mandates of certain services, including provincial ODT programs.

First Nations, Inuit, and Métis patients with organ failure face excessive barriers to transplantation. Organ donation and transplantation programs are urban-centric, limiting access to FNIM patients living in rural, reserve, remote, and northern (RRRN) communities who already face inadequate access to local primary and acute care.^34,35,37^ First Nations, Inuit, and Métis patients experience greater challenges adhering to medical appointments due to geographical remoteness among other factors, with such nonadherence considered a relative contraindication to transplantation. Inequities in transplantation are masked by a paucity of ODT data including the number and rationale for FNIM patients refused placement on waitlists. However, one systematic review demonstrated that FNIM patients with end-stage kidney disease (ESKD) experience longer wait times to receive a kidney transplant and have lower rates of transplant than the general population.^38^

In drafting and passing HOTDA, Nova Scotia moved forward without formal consultation of FNIM groups.^34,37^ Though Canadian governments do not have a legal duty to consult FNIM populations before passing healthcare legislation, as per the United Nations Declaration on the Rights of Indigenous Peoples (UNDRIP), Indigenous peoples have the right to “free, prior and informed consent before adopting and implementing legislative or administrative measures that may affect them.”^39^ The opinions of FNIM individuals on deemed consent are postulated to be influenced by culture, belief systems, and mistrust in the healthcare system. Nevertheless, knowledge of ODT and deemed consent legislation is lacking among the FNIM population and opinions were not established prior to enactment. There is concern that deemed consent will increase the number of FNIM donors without addressing inequities in access to transplantation such that FNIM patients will not see benefit. Government investment in formal FNIM stakeholder representation may allow these and other concerns from FNIM individuals to be appropriately addressed in ODT policy development.

### Big Data

Within Canada, public health databases, including for kidney replacement therapy and transplantation, are managed by several entities (Table 2). Summary statistics from the Canadian Organ Replacement Register (CORR) demonstrated that more than 2700 SOTs occurred in Canada in 2021, with almost all being single organ type and kidney transplants by far the most common.^40,41^ After a slight increase in the early 2010s, deceased kidney donation rates have remained relatively stable across provinces, though with an increase in the proportion of donation after cardiac death (DCD) from approximately 15% to 30%. Most recent CORR reports from 2021 included data from Québec for the first time since 2015 after rectifying privacy concerns, and data from Nova Scotia is now being reported separately from the other Atlantic provinces. The CORR faces ongoing challenges owing to voluntary data collection with waning engagement, and its future role remains unclear.

**Table 2.** Canadian entities responsible for overseeing the management of public health data, including for kidney replacement therapy and transplantation.

Entity	Function
Canadian Institute for Health Information (CIHI)	• Oversees a broad range of health system databases, measurements and standards, together with evidence-based reports and analyses • Partnered with various pan-Canadian healthcare organizations, Statistics Canada, Public Health Agency of Canada, First Nations Health Authority, and Health Data Research Network Canada to provide accurate, comparable, and unbiased information on which to base policies and decisions that influence health^a^
Canadian Organ Replacement Register (CORR)	• National database that captures nearly 600 data elements including demographics, cause of end-stage organ failure, comorbidities, treatment-specific information (e.g., modality of KRT), and SOTR outcomes (e.g., graft failure and mortality) • Generates annual reports on KRT, solid organ donation, and extra-renal SOT, with center-specific reports serving a feedback and quality assurance function with comparative provincial and pan-Canadian statistics^b^
Pan-Canadian ODT data and performance reporting system	• Project lead by CIHI and Canada Health Infoway, funded by Health Canada, to support improvements in ODT access, care, outcomes, and research initiatives • Provides technology solutions for robust data collection, system integration, and pan-Canadian data and system-level performance reporting^c^
Canadian Blood Services (CBS)	• Independent not-for-profit organization that provides blood, plasma, transfusion, and stem cell registry services across provinces and territories except Québec • Facilitates SOT for patients who are difficult to match with a donor^d^

*Note.* CIHI = Canadian Institute for Health Information; KRT = kidney replacement therapy, i.e. dialysis and kidney transplantation; SOTR = solid organ transplant recipients; SOT = solid organ transplantation; ODT = organ donation and transplantation;.

a Canadian Institute for Health Information. About CIHI, https://www.cihi.ca/en/about-cihi (2022, accessed November 29, 2022).

b Canadian Institute for Health Information. Canadian Organ Replacement Register, https://www.cihi.ca/en/canadian-organ-replacement-register-corr (2022, accessed November 29, 2022).

c Canadian Institute for Health Information. Pan-Canadian organ donation and transplantation (ODT) data and performance reporting system project, https://www.cihi.ca/en/pan-canadian-organ-donation-and-transplantation-odt-data-and-performance-reporting-system-project (2022, accessed November 29, 2022).

d Canadian Blood Services. About Canadian Blood Services, https://www.blood.ca/en/about-us (2022, accessed November 29, 2022).

**Figure 3.**
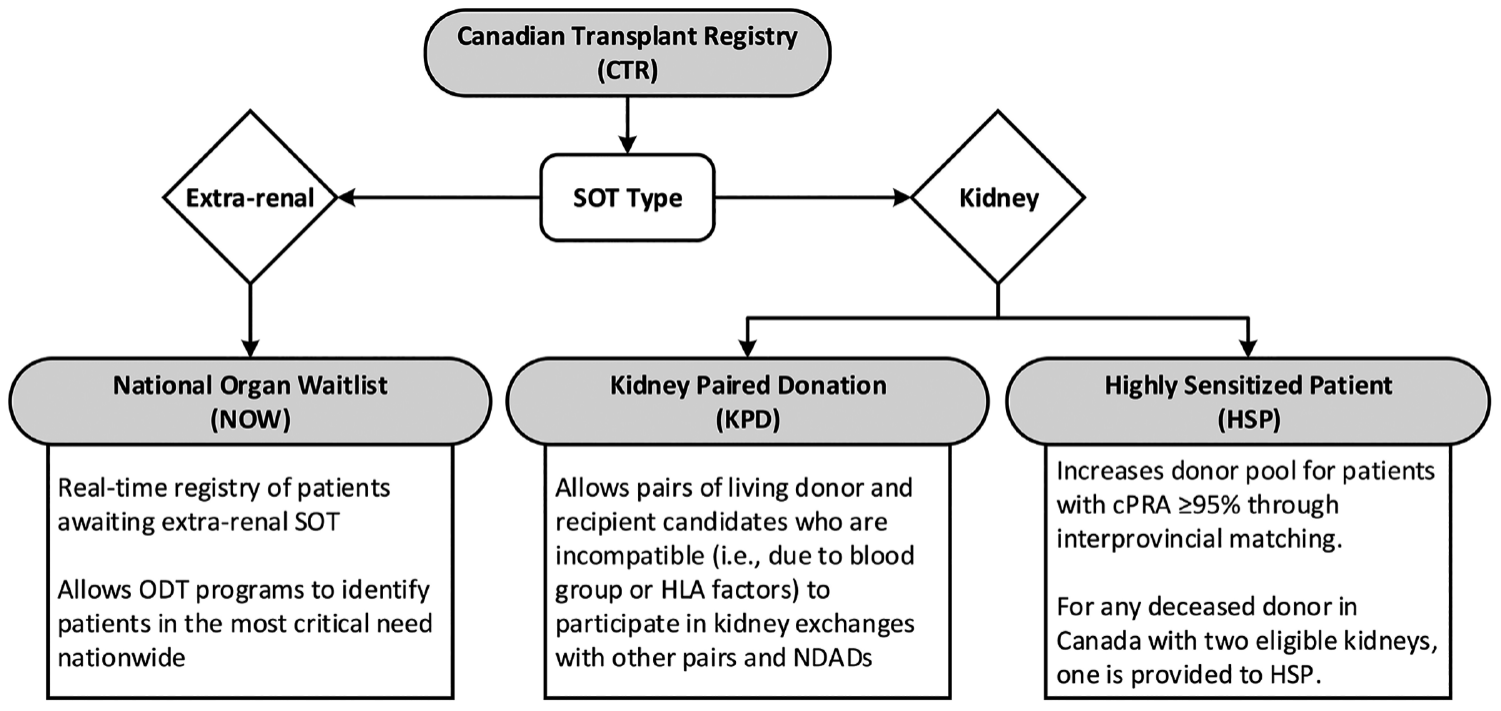
Structure of the CTR, itself comprised of 1 extra-renal SOT registry and 2 kidney transplantation registries.^43^ *Note.* CTR = Canadian Transplant Registry; SOT = solid organ transplantation; ODT = organ donation and transplantation; HLA = human leukocyte antigen; NDAD = nondirected anonymous donor; cPRA = calculated panel reactive antibody; HSP = Highly Sensitized Patient.

The Canadian Transplant Registry (CTR) is a web-based program developed by Canadian Blood Services (CBS) to promote interprovincial sharing of organs and is itself composed of 3 distinct registries (Figure 3).^42^ Since inception, the Kidney Paired Donation (KPD) program has facilitated over 900 kidney transplants in Canada averaging 80 per year. In response to a sharp decline in donors at the start of the COVID-19 pandemic, KPD moved away from mandating that living donors travel to the corresponding recipient’s ODT program and instead started shipping kidneys between centers. While only 6.8% of KPD kidneys between 2009 and 2019 were shipped, that increased to 85.7% in 2021, corresponding to a record 105 KPD transplants including 37 NDADs. The Highly Sensitized Patient (HSP) program has facilitated interprovincial kidney transplantation for greater than 50% of enrolled patients, corresponding to approximately 120 transplants annually. Canadian Transplant Registry has supported quality improvement projects (e.g., optimizing match cycle frequency in KPD, and prioritizing calculated panel reactive antibody [cPRA] ≥99% in HSP), but was not designed to collect outcome data.

The Pan-Canadian ODT Data and Performance Reporting System Project aims to realize a longstanding, overarching goal within the transplant community: to decrease the number of patients on transplant waitlists and deaths while waitlisted through organizing waitlist data and minimizing missed donation opportunities.^44^ The project engaged stakeholders (e.g., clinicians; researchers; ministries of health; and patients, families, and donors [PFDs]) using a modified-Delphi approach to define and prioritize performance indicators, including both general outcomes for all SOTs (e.g., graft survival, patient survival including waitlist mortality, and rejection rates) and organ-specific indicators (e.g., time between dialysis start and transplant referral, and pre-emptive kidney transplant rate).^45^ In parallel, the project is defining minimum standards for data sets required to adequately assess these indicators. iTransplant software (Transplant Connect; Los Angeles, California) is designed to minimize the burden of point-of-care data collection and eliminate antiquated home-grown, paper-based solutions requiring repeat manual entry.^46^ A nation-wide database is required to integrate, store, and analyze such data; this falls beyond the capabilities of CORR and CTR, and unfortunately, information technology infrastructure limitations continue to impede such endeavors.

### Xenotransplantation

Xenotransplantation offers to improve organ supply by recovering organs from nonhuman donors, particularly pigs. To address cross-species immunological incompatibilities and prevent rejection, pigs must be genetically modified. Specific porcine genes are deleted (e.g., targets for innate immunity) while human genes are added (e.g., human complement inhibitor genes, anticoagulant genes, and immunosuppressive and anti-inflammatory genes) to overcome incompatibilities.^47,43^ Pig growth receptor genes are also deleted to ensure appropriate graft size. Xenotransplantation research and development is largely industry funded, with Revivicor, Inc. having raised more than 400 genetically modified pigs to date that have maintained normal growth, fertility, and health in a pathogen-free facility.^48^

Complex xenoantibody testing is required during various stages of xenotransplantation. Preformed IgG and IgM pig antibodies in recipients must be measured during the pretransplant phase.^47^ Since about 1% of naturally occurring human antibodies are directed against α-GAL, felt to be a driving force behind xenotransplant rejection, galactosyltransferase gene knockout (GTKO) pigs lacking α-GAL expression were developed. Human leukocyte antigen (HLA) antibodies have previously been demonstrated to cross-react with swine leukocyte antigens (SLA), which may cause hyperacute or acute rejection; however, a review of more recent studies found that relatively few HSPs have HLA antibodies that cross-react with SLA.^49^ Furthermore, introducing site-specific SLA mutations may minimize or eliminate cross-reactivity.^50^ Xenotransplantation may therefore be of particular benefit for HSPs, with pre-emptive plasma exchange to lower the recipient’s antibody burden and lymphocyte and nonlymphocyte (i.e., porcine aortic endothelial cell) CD crossmatching being potential strategies to further reduce the risk of xenograft rejection.

A decedent human model was recently utilized by investigators at New York University in conducting pig-to-human xenotransplants with kidneys (n = 2) and hearts (n = 2) from GTKO pigs.^43,51^ Recipients declared brain-dead by the clinical care team underwent transplantation consisting of the anastomosis of xenograft vessels to the femoral artery and vein, with the xenograft left outside the body on the thigh for ease of direct observation and serial biopsies. These 2 decedent kidney transplants resulted in excellent xenograft urine output and doubling of estimated glomerular filtration rate (eGFR) by the end of the study period (54 hours after reperfusion). Biopsies obtained at the end of the study period did not demonstrate evidence of hyperacute rejection, disseminated intravascular coagulation (DIC), hyperinflammation, excessive cytokine release (IL-2, IL-6, IL-10, IL-13), or complement dysregulation. Additional histological assessment demonstrated glomerulitis with less marked tubulointerstitial inflammation equivocal or suspicious for early antibody mediated rejection (AMR) and acute tubular necrosis.^52^ There remains much to be learned about the adaptive immune responses to xeno-antigens that longer-term decedent studies may elucidate, but these recent advances are promising.

### Noninvasive Diagnostics

Smoldering immune activation in clinically stable kidney allografts can lead to fibrosis and progressive graft failure.^53^ Noninvasive diagnostics using urine and blood samples may replace the need for protocol biopsies and provide earlier detection of subclinical rejection. The Q-Sant test (NephroSant; Brisbane, California) utilizes 6 urine biomarkers (total protein, cell free DNA [cfDNA], mitochondrial-cell-free DNA [m-cfDNA], C-X-C motif chemokine ligand 10 [CXCL10], clusterin, and creatinine) and artificial intelligence to calculate a personalized Q-score as a measure of subclinical and clinical acute rejection.^54^ Q-scores correlate to pathological findings (i.e., Banff acute rejection scores) and have been validated in both adults and children.^55^ However, further development is required to be able to distinguish acute rejection from other injuries such as ischemia-reperfusion injury (IRI).

Cell-free DNA (cfDNA) is a potentially useful serum marker of subclinical rejection.^56^ The ADMIRAL study was a retrospective, multicenter study that monitored donor-derived cfDNA (dd-cfDNA) among 1092 kidney transplant recipients over a 3-year period.^57^ Although elevated dd-cfDNA was associated with both de novo donor-specific antibodies (DSAs) and eGFR decline, results were likely confounded by graft dysfunction at the time of assessment. To address this, a subsequent retrospective observational single-center study focused on dd-cfDNA surveillance in stable patients with normal allograft function (i.e., creatinine less than 1.5 mg/dL; absence of DSAs; and no history of clinical rejection) over a median follow-up period of 1.5 years.^58^ Although elevated dd-cfDNA was a predictor of rejection, most patients with elevated dd-cfDNA remained clinically stable. The utility of cfDNA monitoring alone in the setting of stable allograft function remains unclear.

TruGraf (Eurofins Transplant Genomics; Framingham, Massachusetts) testing uses DNA microarray technology to measure 120 genes previously identified to be differentially expressed among patients with and without histological evidence of rejection.^59-61^ TruGraf is therefore another potential tool for identifying subclinical rejection, with surveillance testing corresponding well to histological findings including therapeutic response. As TruGraf appears to outperform dd-cfDNA in the diagnosis of subclinical cellular rejection, and dd-cfDNA appears to outperform TruGraf in the diagnosis of subclinical AMR, combining these 2 tools may be beneficial.^62,63^ Although such noninvasive diagnostics have been implemented in the United States and other countries, implementation in Canada has been limited due to cost and other considerations.

### Cutting-Edge Technologies

As the use of more marginal kidney allografts expands to match rising demand, novel methods are required to optimize allograft preservation.^64^ The current standard of static cold storage (SCS), whereby kidneys are stored on ice between 2°C and 6°C, is associated with cold injuries that contribute to delayed graft function and graft failure.^65^ Normothermic machine perfusion (NMP; ie, 35°C-37°C) and subnormothermic machine perfusion (SNMP; ie, 20°C-22°C) have therefore been postulated to offer improved graft outcomes. Hydrogen sulfide (H_2_S) has diverse cytoprotective effects, fuelling the development of perfusion solutions containing H2S donor compounds.^66^ AP39 is one such compound and has previously been associated with decreased IRI for kidney allografts in SCS.^67,68^ Investigators in London, Ontario combined these interventions, demonstrating that ex vivo porcine kidneys subject to SNMP with AP39-supplemented autologous blood experienced improved urine output and decreased expression of injury and pro-survival genes compared to kidneys subject to SCS or standard SNMP with blood alone.^69^ In a subsequent in vivo pilot trial, patients undergoing DCD kidney transplantation with organs subject AP39-supplemented perfusion solution experienced decreased delayed graft function, higher urine output, and lower proteinuria as compared to standard solution.^70^ Modified perfusion temperatures and AP39-supplemented perfusion solutions are inexpensive methods to potentially transform organ preservation.

**Figure 4.**
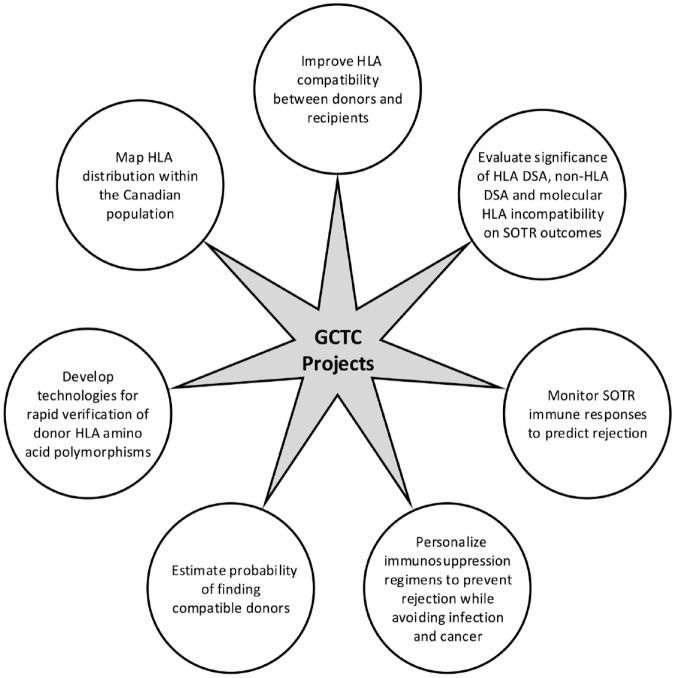
Goals of various projects supported by the GCTC. *Note.* GCTC = Genome Canada Transplant Consortium; HLA = human leukocyte antigen; DSAs = donor-specific antibodies; SOTR = solid organ transplant recipient.

The Genome Canada Transplant Consortium (GCTC) aims to optimize SOTR outcomes through policy-making and the development of tools for use in various aspects of SOT (Figure 4).^71^ The GCTC has been examining the legal, ethical, societal, and economic considerations of introducing molecular compatibility tools into clinical practice.^72^ With stakeholders having expressed concern regarding the implication of molecular compatibility tools on inequities in SOT access, the Kidney Transplant Simulation model was developed by GCTC to allow comparisons between present deceased donor allocation systems and proposed future systems to further support ODT programs in decision-making regarding molecular compatibility implementation.

## Limitations

We chose to summarize presentations within the core program of the conference relevant to clinical care for SOT in general and kidney transplantation specifically, recognizing that kidney transplants represent the vast majority of SOTs in Canada. We were unable to capture all presentations and topics at the meeting due to the sizable quantity and variety. Recordings were available for only a portion of presentations, impeding retroactive inclusion of presentations. Topics ultimately excluded from this summary include those in pathology including Banff Classification updates; those unique to extra-renal SOT; as well as numerous abstract and poster presentations, allied health provider forums, and business meetings. A portion of the material was presented by speakers prior to peer-review or publication and therefore validity could not be formally assessed.

## Implications

Transplantation in Canada is evolving. COVID-19 continues to pose a threat to SOTRs though outcomes have improved with the development of novel treatment and prevention strategies. Infection control protocols have spawned efficient virtual methods of research and patient care. Optimizing reimbursement programs and making evaluations more efficient may overcome the many disincentives faced by potential living kidney donors. Upcoming consensus expert recommendations offer to standardize ODT programs nationally and internationally, while experiences from the implementation of deemed consent in Nova Scotia may provide further insight to guide ODT reform. In Canada, FNIM patients have been identified as a population requiring distinct consideration in ODT owing to poorer health outcomes and limited access to transplantation compared to the general population. Ongoing development of data repositories may improve patient care delivery while allowing for establishment of national benchmarks on which ODT programs can base quality improvement. Additional advancements in xenotransplantation, noninvasive diagnostics for identification of subclinical rejection, graft preservation techniques, and molecular compatibility may further contribute to improvements in both access to transplantation and long-term SOTR outcomes.
